# Embracing innovation and advancing care: integrating learning health system principles into Inova Schar Heart and Vascular

**DOI:** 10.3389/fcvm.2024.1409303

**Published:** 2024-10-24

**Authors:** Christopher M. O’Connor, Carolyn M. Rosner, Andrew Gill, Alan M. Speir, Richard F. Neville

**Affiliations:** Divisions of Cardiology, Cardiac and Vascular Surgery, Inova Schar Heart and Vascular, Falls Church, VA, United States

**Keywords:** innovation, learning health care system, cardiovascular care and outcomes, systems of care, integration of care

## Abstract

Inova Schar Heart and Vascular has an unwavering commitment to delivering excellent cardiovascular care and has integrated principles of a learning health care system to develop our system of continuous process improvement and innovation. A learning health system integrates its internal experiences with external research to enhance patient outcomes, support the discovery of new treatments and care pathways, and deliver safer, more efficient, and more personalized care. Leveraging learning across health systems maximizes the impact, allowing cardiovascular teams to gain insights into the effectiveness of different treatment strategies. In this Frontiers in Cardiovascular Medicine compendium of articles, the team at Inova describe the spectrum of research and educational activities that have contributed to our progress as a learning cardiovascular health system and support our journey to deliver excellent cardiovascular care.

Inova Schar Heart and Vascular has an unwavering commitment to delivering excellent cardiovascular care and has integrated principles of a learning health care system to develop our system of continuous process improvement and innovation.

A learning health system integrates its internal experiences with external research to enhance patient outcomes, support the discovery of new treatments and care pathways, and deliver safer, more efficient, and more personalized care (1). The National Academy of Medicine has extensively studied learning health systems and identified several key benefits, including enabling the collection and utilization of data on a large scale to analyze trends and patterns in cardiovascular diseases. This learning can lead to the identification of specific risk factors, early detection of disease or patterns of deterioration of clinical conditions, and ultimately the development of preventative or mitigating strategies.

Leveraging learning across health systems maximizes the impact, allowing cardiovascular teams to gain insights into the effectiveness of different treatment strategies. The collaborative sharing of best practices, evidence-based guidelines, and research findings through presentations and publications fosters continued innovation and adoption of new technologies, ultimately improving the quality of care provided to patients (1–3) (Figure 1).

**Figure 1:**
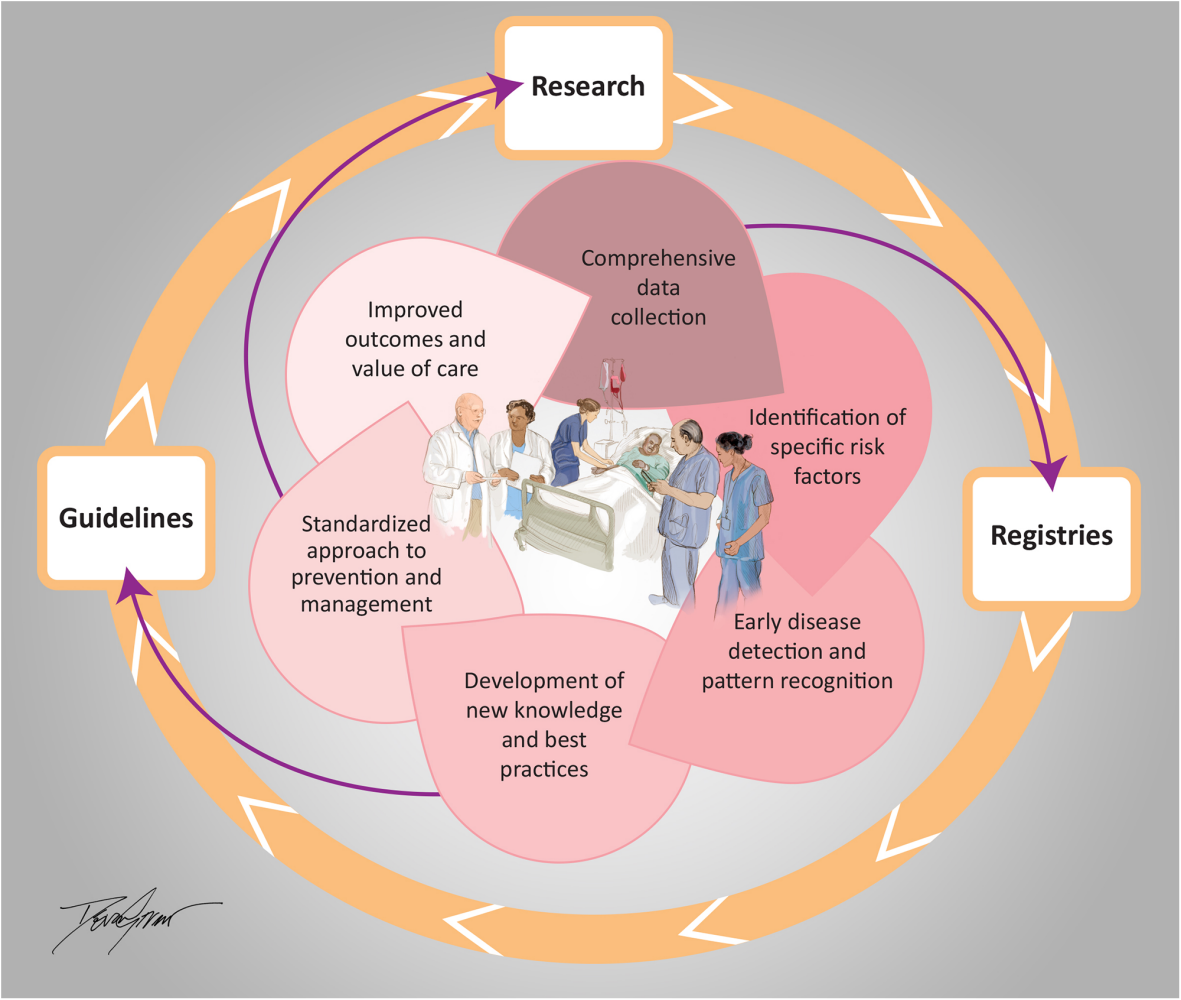
Inova schar heart and vascular integrated learning health care system. Illustrated by Devon Stuart (2024).

A culture of research and education within a learning health system environment supports a drive to excellence summarized in this *Frontiers in Cardiovascular Medicine* compendium of articles. Our team members describe the spectrum of research and educational activities that have contributed to our progress as a learning cardiovascular health system and support our journey to deliver excellent care. A few examples of learning health system projects that have been implemented at Inova Schar Heart and Vascular include:

• The Inova Cardiogenic Shock Program, developed to address the high mortality seen in patients in Northern Virginia presenting with cardiogenic shock (CS) due to heart attack and heart failure, has used the data collected in the Inova Shock Registry to identify key opportunities for care transformation and developed processes to optimize care delivery, reduce variation, and improve outcomes. Hospitals within and outside the health system adopted our standardized clinical care pathways for identification and management of CS and as patients with CS were cared for and data was added to the registry, the team was able to identify additional important factors, such as unique shock etiologies and disparities in care, associated with higher mortality (4, 5). This focus has resulted in improved survival for many patients with CS and our experience developing and sustaining a protocol-based approach to CS care is discussed by Mehta, Sinha, et al. in this special compendium of articles (6).
• Similarly, protocols developed to streamline the management of aortic emergencies (ruptured aneurysms and dissections) brought together key stakeholders across the health system, including patient transport, emergency services, the operating room, the catheterization lab, nursing, residents, and multidisciplinary attending staff and resulted in standardized care, improved efficiency and importantly, saved lives in this high-risk population. The process and outcomes are regularly evaluated and discussed at scheduled debriefing sessions to encourage ongoing process improvement.
• Another example of learning arising from registry participation is in our vascular surgery program, which utilized important benchmarking information from the Vascular Quality Initiative (VQI) registry to improve carotid endarterectomy outcomes by reducing operator variation, particularly among those with low volumes (7).
• Inova Schar Heart and Vascular was also a founding member and leader in the Virginia Cardiac Services Quality Initiative, a consortium of 18 Virginia Hospitals and 14 cardiac surgical practices and cardiology specialties formed with the goal of improving the outcomes and value of cardiac procedures in the state of Virginia through sharing and reviewing data to learn and implement best practices. One of the important initiatives that came from this was development of new protocols which allowed the Inova team to reduce the blood transfusion needs of patients undergoing bypass surgery without compromising clinical outcomes (8).
• Training programs enhance lifelong learning and the development of our cardiovascular fellowship program facilitated expanding knowledge in both learners and providers and led to improved clinical outcomes in the Cardiac Intensive Care unit. Our cardiology fellows are integral members of the multidisciplinary CV teams and have led and participated in many of the contributions included in this publication (6, 9–11).
• Engaging all clinicians is an essential step as a learning health care system focused on delivering excellent cardiovascular care, and Advanced Practice Providers (APPs) lead several of our key initiatives, including an outpatient urgent heart failure clinic (UHFC) (12). The UHFC provides IV diuretics and is a critical part of a successful system of care for the ambulatory management of decompensated heart failure, described in this collection (13).
• Equipping clinicians for leadership roles through the development and implementation of an integrated leadership training academy for physicians and APPs has been another key initiative that yielded increased engagement and effectiveness needed in a learning health system and led to greater leadership responsibility (14).
• The “Heart Failure Collaboratory” is a public-private partnership developed between Inova Schar Heart and Vascular and the U.S. Food and Drug Administration (FDA) and brings stakeholders together to rethink both the development and implementation of clinical research for patients with heart failure, including strategies to encourage increased utilization of guideline based medical therapy (11). Inova aspires to be a leader in this by developing electronic health record-based strategies to engage clinicians to utilize guideline-based treatment for their patients with heart failure, via strategic prompts aimed at increasing appropriate medication prescription and referral for advanced heart failure, structural heart and electrophysiology care (15).

FDA Commissioner Dr. Robert Califf has highlighted the significance of learning health systems and in his writings, he emphasizes the need to bridge the gap between clinical research and practice. By integrating research into routine clinical care, cardiovascular health systems can generate real-world evidence, which can be used to inform treatment decisions and improve patient outcomes (15).

In summary, learning health system principles promote the integration of research, practice, and data analysis and elevate clinical care and outcomes. (Figure 1) This integration can lead to the discovery of new treatments and protocols, provide more personalized care, and facilitate excellent cardiovascular care and patient outcomes.

The main concepts of the Integrated Learning Health Care System at Inova Schar Heart and Vascular, with integration of research, registries and guidelines to inform, develop and implement strategies to deliver excellent cardiovascular care and outcomes.

## Data Availability

The original contributions presented in the study are included in the article/Supplementary Material, further inquiries can be directed to the corresponding author.
